# Outcomes and Complications of Total Joint Arthroplasty in Heart Transplant Recipients: A Systematic Review

**DOI:** 10.3390/jcm15176794

**Published:** 2026-09-01

**Authors:** Alexandros Koskiniotis, George A. Komnos, Antonios Koutalos, Michael Hantes, Theofilos Karachalios, Sokratis Varitimidis, Nikolaos Stefanou

**Affiliations:** Department of Orthopaedic Surgery, Faculty of Medicine, University of Thessaly, 3 Panepistimiou St, Biopolis, 41500 Larissa, Greeceakoutmed@gmail.com (A.K.);

**Keywords:** heart transplantation, total shoulder arthroplasty, total knee arthroplasty, total hip arthroplasty, systematic review

## Abstract

**Background**: Heart transplantation has evolved into a life-saving intervention for thousands of patients with end-stage disease worldwide. Advances in surgical techniques and immunotherapy have increased life expectancy in this specific patient group, resulting in the emergence of degenerative diseases such as osteoarthritis. Concurrently, due to the frequent administration of corticosteroids to transplant recipients, the rates of joint osteonecrosis remain high. This review aims to evaluate the final functional outcomes as well as the complications of patients with a history of heart transplantation who underwent total hip, knee, or shoulder arthroplasty. **Methods**: A systematic review of the literature was conducted according to PRISMA guidelines. The primary objective of the study was the improvement of various functional scores at the final clinical follow-up, as well as the recording of postoperative complication rates. **Results**: A total of 19 retrospective studies were included in the review, including small case series. Among the studies specifically evaluating functional outcomes, patients demonstrated statistically significant improvement in final clinical scores and quality of life. However, regarding complication rates, 12 out of the 19 articles identified a higher ratio compared to the general population, involving either systemic (e.g., renal failure), musculoskeletal (periprosthetic fracture, infection), or cardiac-related adverse events concerning the graft. **Conclusions**: According to the data analyzed in this systematic analysis, total arthroplasty of major joints demonstrates consistent potential to significantly improve the daily life and functional status of transplant patients. However, evidence regarding overall safety remains heterogeneous.

## 1. Introduction

Total joint arthroplasty (TJA) has evolved into a revolutionary intervention for restoring quality of life in patients suffering from severe, chronic arthritis. The continuous progress of surgical techniques and implant design, coupled with an aging population and excellent clinical outcomes, has led to a significant increase in procedural volume worldwide, currently estimated at over 2 million cases annually [1]. Projections indicate that this volume will rise even more, with increases of approximately 120–200% expected by 2050 [2].

Parallel advancements in vital organ transplantation, regarding both surgical technique and immunosuppressive regimens, have significantly extended the life expectancy of transplant recipients. Unlike in the past, heart transplant patients now frequently live long enough to develop degenerative arthritic changes that severely impact their daily activities and overall quality of life [3]. Indeed, survival rates exceeding 20 years have been reported in over 50% of heart transplant recipients in some series [4]. However, this population is particularly vulnerable to osseous pathology and joint osteonecrosis, often as a consequence of chronic, high-dose corticosteroid therapy.

Consequently, TJA is being increasingly performed in heart transplant recipients, with various series reporting satisfactory results, although unique perioperative challenges remain. Chronic exposure to immunosuppressive agents makes these patients susceptible to infection, poor wound healing, and general systemic compromise [5]. The decision to proceed with TJA should carefully balance the potential for improved quality of life against the risk of complications, which may be life-threatening in this special category of patients.

The current evidence regarding TJA in this population is largely limited to retrospective series, mostly due to the rarity of these specific patients. Given the scarcity of high-level evidence, a systematic review is necessary to clarify the safety and efficacy of joint reconstruction in this specific group; this information will also help in the accurate preoperative counseling of candidate patients. The aim of this systematic review is to evaluate whether heart transplant recipients can safely undergo TJA and to delineate the expected outcomes and complications. Additionally, this review seeks to compare these parameters against those of the general TJA population and recipients of other solid organ transplants.

## 2. Materials and Methods

Search Strategy and Guidelines: This systematic review was conducted in accordance with the Preferred Reporting Items for Systematic Reviews and Meta-Analyses (PRISMA) guidelines (Supplementary Materials). A systematic literature search was performed using the PubMed, Scopus and Web of Science databases to identify relevant studies published between 1 January 2000 and 1 November 2025. Studies published prior to 2000 were excluded to ensure that the analysis included modern advancements in arthroplasty techniques and immunosuppressive regimens. The search algorithm utilized combinations of the following terms across all three databases (adapted appropriately for PubMed MeSH terms and Scopus/Web of Science Boolean operators): (heart transplant OR cardiac transplant) AND (hip replacement OR knee replacement OR shoulder replacement OR hip arthroplasty OR knee arthroplasty OR shoulder arthroplasty OR “total hip” OR “total knee” OR “total shoulder”). A formal protocol was not prepared prior to our review, and the study was not prospectively registered on platforms such as PROSPERO. We acknowledge this as a deviation from ideal systematic review methodology, which may introduce a potential for selection or reporting bias.

Study Selection: The initial search yielded 231 articles. Screening and selection were performed manually by two independent reviewers without the use of automation tools. Differences regarding study inclusion were resolved through consultation with a third senior reviewer.

Inclusion and Exclusion Criteria: Studies were included if they met the following criteria: (1) patient population included heart transplant recipients undergoing major joint reconstruction (hip, knee, or shoulder) and (2) the study evaluated functional outcomes, quality of life, or postoperative complication rates. Exclusion criteria were: (1) articles published in languages other than English, (2) studies with unavailable full texts and (3) systematic reviews, conference abstracts, review articles, and expert opinions.

**Figure 1:**
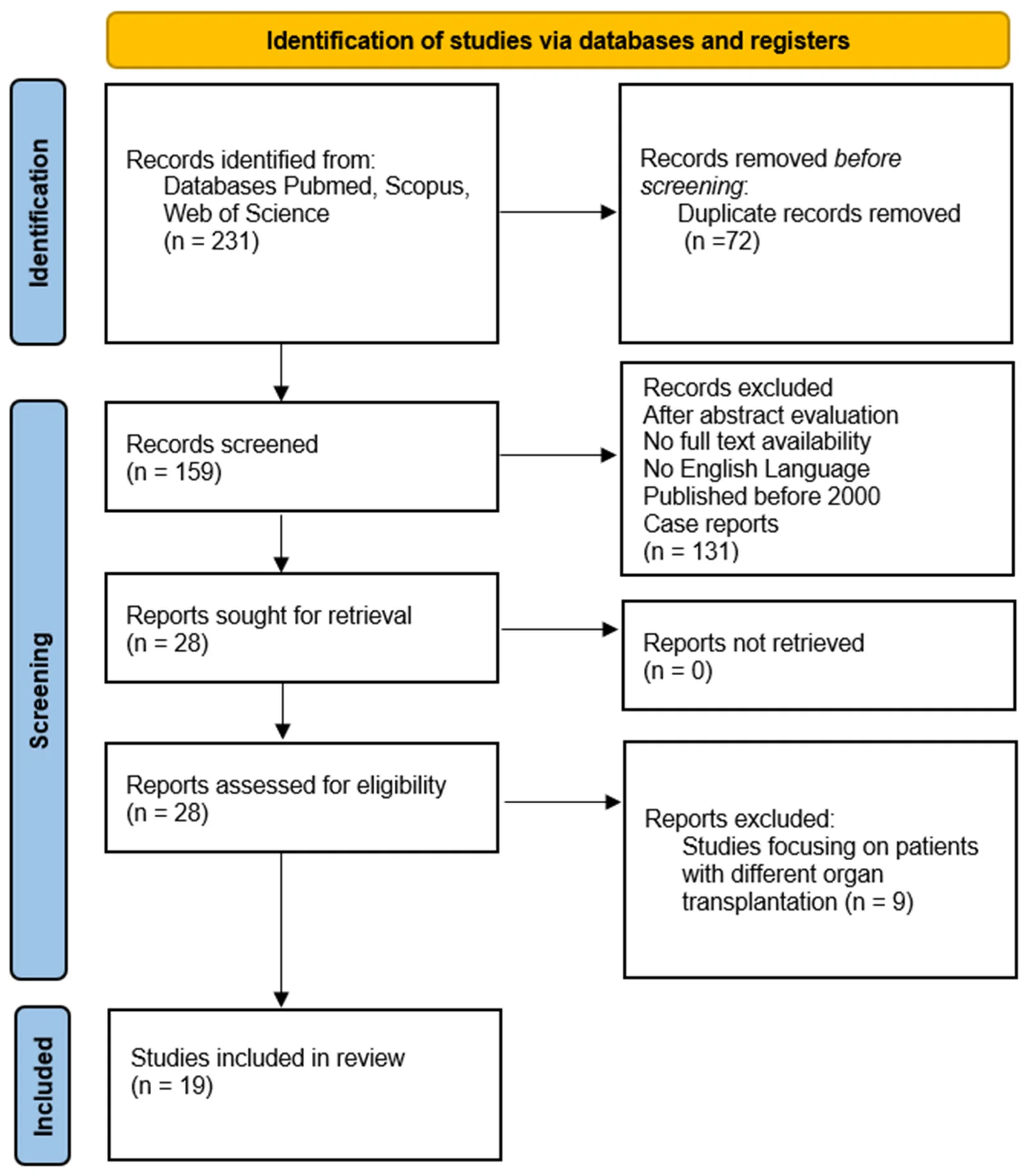
PRISMA Flowchart.

Data Extraction: Following the final selection, data were systematically extracted and analyzed. In studies reporting on mixed cohorts of solid organ transplant recipients, data specific to heart transplant patients were isolated for analysis. A detailed summary of the selection process is provided in the PRISMA flowchart in Figure 1, and the study characteristics are categorized in Table 1.

### Data Synthesis

A quantitative meta-analysis was deemed inappropriate and therefore not performed due to profound methodological and clinical heterogeneity across the included literature. Specifically, this heterogeneity manifested in wide variations in study design (ranging from small institutional case series to large retrospective national database queries), inconsistent definitions and reporting metrics for clinical outcomes, and highly variable follow-up durations. Furthermore, the inclusion of several large databases introduced a high risk of overlapping patient populations, which would violate the assumption of independence required for pooled quantitative synthesis. For this same reason, outcome-specific quantitative syntheses for discrete endpoints such as mortality, periprosthetic joint infection, revision, or readmission were considered but ultimately rejected. The varying definitions of these complications across studies, the mixture of adjusted effect estimates and raw proportions reported across the literature, and the inability to isolate independent patient samples among the aggregated administrative databases meant that any statistical pooling would carry an unacceptably high risk of bias. The control groups also varied significantly across studies, with some utilizing non-transplant cohorts and others utilizing recipients of different solid organs. Consequently, a qualitative synthesis was chosen as the only appropriate methodological choice to accurately reflect the available evidence. The extracted data were grouped thematically based on the primary outcomes of interest: functional improvements, implant survivorship and revision rates, patient mortality, and general complication rates.

Quality Assessment/Risk of Bias: The methodological quality of the included studies was evaluated by two reviewers using the Methodological Index for Non-Randomized Studies (MINORS) criteria, which was specifically designed for assessing the quality of non-randomized studies. Any discrepancies in scoring were resolved through discussion with a third senior reviewer. The results of the MINORS quality assessment are presented in Table 2.

## 3. Results

A total of 19 studies published between January 2000 and November 2025 met the inclusion criteria (Table 1). Due to the relatively low incidence of total joint arthroplasty in this population, all included studies were retrospective in design, a factor which limits the overall level of evidence. Of the 19 studies, only three exclusively investigated heart transplant recipients. The remaining 16 studies evaluated broad solid organ transplant (SOT) cohorts (including kidney, lung, heart, and liver), from which cardiac-specific data were manually extracted.

The cumulative estimated cohort across all 19 studies represents over 1000 patients who underwent major joint reconstruction. However, it is critical to classify these studies based on data availability. Four large administrative database studies (Navale, Klika, Cavanaugh, Quinlan) aggregated heart transplant patients into general “Solid Organ Transplant” or “Other” categories, meaning that isolated cardiac data could not be definitively extracted. When excluding these aggregated database studies, the recalculated, confirmed sample size of isolated heart transplant recipients explicitly evaluated in the remaining literature is 1152 patients. The majority of procedures were total hip arthroplasties (THAs, *n* ≈ 730) and total knee arthroplasties (TKAs, *n* ≈ 400), with only a small minority involving the shoulder. The mean age of the cohort was approximately 58 years, significantly younger than the mean age of the general population undergoing TJA for degenerative joint disease [25]. Furthermore, it must be acknowledged that the inclusion of multiple large, national retrospective databases spanning similar timeframes introduces a high probability of overlapping patient populations. Therefore, the cumulative sample size should be interpreted as an aggregate of procedural data points rather than entirely unique, individual patients.

While osteoarthritis was the most common indication for surgery, osteonecrosis (avascular necrosis) accounted for a high percentage of cases (35–60%). This high prevalence of osteonecrosis is likely attributable to chronic corticosteroid-based immunosuppression [26].

The focus of the included literature varied: ten studies primarily evaluated perioperative complications (e.g., infection, transfusion requirements, mortality, readmission), six focused on functional outcomes, and three evaluated both domains equally. Regarding comparative analysis, ten studies used a non-transplant control group, five compared outcomes across different solid organ transplant types, and four were non-comparative series. Follow-up duration varied significantly based on study design, ranging from immediate in-hospital outcomes to long-term follow-up exceeding 17 years.

Before detailing the specific clinical outcomes, it is helpful to frame these findings within the context of their methodological quality. Based on our MINORS assessment the included literature presents a mix of ‘Good’ and ‘Fair’ quality evidence. Naturally, clinical trends supported by ‘Good’ quality studies afford a higher degree of confidence. Conversely, findings driven primarily by ‘Fair’ quality data carry a higher potential for bias and should be interpreted with caution. Notably, studies reporting functional outcome scores were predominantly small case series or cohorts classified as ‘Fair’ quality, whereas studies reporting complication, mortality, and revision data from large databases achieved ‘Good’ quality ratings more frequently. Readers should therefore weigh the functional-outcome findings below with somewhat greater caution than the complication and revision data that follow.

### 3.1. Outcomes and Functional Results

Functional improvements following arthroplasty in heart transplant recipients were reported as excellent, often equal to those seen in non-transplant populations. In the hip, León et al. [8] reported a substantial improvement in the mean Harris Hip Score (HHS) from 33.5 preoperatively to 83.8 postoperatively, with patients achieving significant pain relief, excellent hip mobility and functional independence. Similarly, Leonard et al. [7] observed high postoperative scores, reporting a mean HHS of 88.6 for osteoarthritis patients and 71.8 for those with osteonecrosis, noting that 80% of hips were entirely pain-free at final follow-up, similar to Cosic et al.’s series [15]. Ledford et al. [17] recorded the most significant improvement among all solid organ transplant (SOT) subtypes, with heart transplant recipients demonstrating a mean HHS increase of over 50 points. As for the shoulder, outcomes were generally favorable, though slightly inferior compared to healthy controls. Rizk et al. [19] noted that while heart transplant patients achieved significant functional outcomes, their postoperative UCLA scores and active elevation were statistically lower than those of non-transplant controls. On the other hand, Hatta et al. [9] found no significant difference in ASES scores or range of motion between transplant recipients and controls at a mean follow-up of 39 months, suggesting that immunosuppression does not compromise soft tissue recovery or joint mechanics.

### 3.2. Implants Survivorship and Revision Rates

Implant survivorship in heart transplant recipients appears durable, at least at mid-term follow-up, though revision rates varied by study. In an analysis of 18 THAs, Chalmers et al. [12] reported a 5-year implant survivorship of 94.4%, without any revision for aseptic loosening at 100%. This is also supported by Simon et al. [14], who reported a 100% reoperation-free survival rate at 5 years in a cohort of heart transplant patients, which was significantly superior to the lung transplant cohort. However, earlier case series suggested higher instability. Leonard et al. reported a 20% revision rate in their THA cohort at a mean of 7.3 years, primarily driven by aseptic loosening [7]. In large database comparisons, the revision risk appears favorable. Quinlan et al. found that the “non-renal” SOT group (including heart) actually demonstrated a significantly lower odds ratio for all-cause revision (OR: 0.22) compared to non-transplant controls [21]. Conversely, Agarwal et al. noted a 2-year all-cause revision rate of 2.4% in THA, with periprosthetic joint infection accounting for a significant portion of late revisions in their dataset [6].

### 3.3. Mortality and Patient Survival

Mortality outcomes in heart transplant recipients varied significantly based on the follow-up duration and surgical indication. In the immediate perioperative period, death is rare. Palmisano et al. recorded a 0% mortality rate in their case series, while Agarwal et al. observed a low 90-day mortality of 0.65% in a large database cohort [6,18]. However, outcomes at one year showed a trend toward increased risk. Quinlan et al. noted a 1-year mortality rate of 2.83% for non-renal transplant recipients (including heart), which was higher than non-transplant controls (*p* = 0.06) [21].

It is critical to distinguish elective joint reconstruction from trauma settings. Concerning hip fractures, Hsiue et al. evaluated a distinctly different clinical scenario—urgent arthroplasty and hemiarthroplasty for femoral neck fractures—and found a significantly elevated mortality risk (OR: 2.38) for transplant patients compared to the general population [11]. This fracture-related cohort must not be directly equated with elective arthroplasty populations, as the acute trauma setting inherently carries a substantially higher baseline mortality and complication risk, which is further amplified by the patients’ complex immunosuppressed status [11]. Long-term survival is also influenced by the patient’s medical complexity. Brown et al. reported an odds ratio of 7.42 for mortality in solid organ transplant patients compared to controls over a period of at least 5 years [20]. Despite these elevated risks relative to the general population, heart transplant patients often exhibit superior survival compared to other transplant recipients. Wu et al. found heart recipients had significantly lower mortality than lung and kidney recipients [13], and Simon et al. reported a 5-year patient survival of 83.3% for heart recipients, which was quite better than the 68.6% survival seen in lung recipients [14]. Most authors concluded that while perioperative mortality remains low, long-term patient survival is mostly impacted by the underlying comorbidities associated with cardiac transplantation, unrelated to the arthroplasty procedure.

### 3.4. General Complications and Infection

Heart transplant recipients demonstrate a unique complication profile characterized by high medical resource utilization and specific mechanical risks. In terms of systemic issues, Navale et al. and Cavanaugh et al. identified significantly elevated risks of acute renal failure (OR: 4.42) and blood transfusion in heart and solid organ transplant recipients in general [22,23]. Ledford et al. reported the highest transfusion rate (75%) and medical complication rate (deep vein thrombosis, urinary tract infections, cardiac events—50%) among all organ types in their cohort [17], while Wu et al. noted that nearly one-third of heart patients visited the emergency department within 90 days, primarily for medical management such as syncope or rejection workups [13]. Agarwal et al. specifically highlighted cardiac risks, reporting that a high proportion of heart transplanted patients experienced cardiac complications and 24% suffered a myocardial infarction post-surgery [6]. Surgically, the complication profile of the specific patients goes beyond infection. Cosic et al. reported a 76% complication rate in heart and lung recipients, noting intraoperative fractures as a critical risk [15]. Klement et al. confirmed this in total knee arthroplasty, identifying heart transplant recipients as having the highest risk for periprosthetic fracture (OR: 8.87) of any transplant group at 3 months, even though there was no difference at the final follow-up [10]. Other mechanical and soft-tissue complications were also prevalent. León et al. observed a 15.8% rate of heterotopic ossification in their THA cohort, and Leonard et al. noted stiffness requiring manipulation under anesthesia in knee patients [7,8]. Interestingly, despite chronic immunosuppression, several cohorts like Klatt et al. and Palmisano et al. emphasized that while general complication rates are high, infection rates in heart patients were notably low (approximately 0%), in contrast with the higher infection proportions seen in renal and liver cohorts [16,18]. Lastly, Klika et al. reported that patients with a history of solid organ transplantation undergoing TKA had a slightly longer length of stay and were approximately 1.43 times more likely to experience any complication [24].

## 4. Discussion

The global volume of heart transplantation has risen steadily over recent decades, accompanied by stable or improving postoperative mortality rates. Data from US transplant registries and the American Journal of Transplantation indicate an approximate 86% increase in adult heart transplant procedures compared to 15 years ago. Long-term outcomes have similarly improved, with reported 5-year survival rates now exceeding 80% [27]. In Greece, although transplant programs have historically lagged behind those of other European nations regarding absolute procedural volume, recent years have shown significant progress. Improvements in healthcare administration and the organization of the national transplant system have resulted in increased donor availability and a rising number of successful procedures [28].

To our knowledge, this is the first systematic review to exclusively evaluate the outcomes and complications of large joint arthroplasty specifically in heart transplant recipients. Previous literature has mostly categorized solid organ transplant (SOT) recipients as a single high-risk cohort, often failing to differentiate the organ-specific pathophysiological needs and complication profiles associated with each transplant type. Notably, our analysis indicates that among the broader SOT population, heart transplant recipients exhibited some of the lowest rates of mortality and revision. These findings generally align with the trends observed in other systematic reviews. Patel et al. [29], in a review of shoulder arthroplasty in SOT patients, reported no significant differences in functional outcomes, surgical complications, or revision rates compared to non-transplant controls. Similarly, previously published work by Sayed-Noor [30] concluded that despite an elevated theoretical risk profile, TJA remains a safe and highly beneficial procedure for this population. However, the medical complexity of this population is a fact that cannot be discounted. Kim et al. [31] recently demonstrated that transplant recipients experience a higher complication profile regarding readmission, blood transfusion requirements, and medical comorbidities compared to the general population. Finally, regarding periprosthetic joint infection (PJI), a primary concern given the necessity of chronic immunosuppression, the current literature does not support a correlation with significantly elevated revision rates for infection, suggesting that the theoretical risk may be overestimated [5].

This review is subject to several limitations, mostly due to the quality and design of the available literature. Because of the profound heterogeneity in patient populations, baseline comorbidities, and surgical indications across the included studies, synthesizing definitive conclusions regarding universal safety is challenging, with precise estimates of long-term complications and mortality remaining uncertain. Firstly, all included studies were retrospective in nature, which limits the level of evidence and introduces a possibility of selection bias. The absence of prospective, randomized trials is attributed to the relative scarcity of heart transplant recipients undergoing TJA compared to the general population, making large-scale prospective trials extremely difficult. Secondly, significant heterogeneity exists across the included studies regarding surgical protocols, immunosuppressive regimens, and outcome reporting. Furthermore, a principal methodological limitation is the absence of a prospectively registered review protocol (e.g., PROSPERO). Without a predefined protocol, there is an increased risk of introducing bias during study selection, data extraction, and outcome reporting, which should be considered when interpreting the certainty of this evidence. A specific limitation concerns our data as well. In order to maximize the sample size and statistical power of our analysis, we included large database studies that aggregated heart transplant recipients into broader “solid organ transplant” or “non-renal transplant” cohorts. This approach inevitably limits the specificity of our conclusions. Outcomes after liver, kidney, lung, and heart transplantation differ substantially regarding required immunosuppressive regimens, baseline comorbidities, cardiovascular risk profiles, infection susceptibility, and long-term survival. While this aggregation obscures heart-transplant-specific physiology to some degree, excluding these large cohorts would have severely compromised the overall data volume of the review. We proceeded with this approach, acknowledging the risk of potential bias. Finally, formal assessment of publication bias was not feasible given this qualitative synthesis; overall evidence certainty is considered limited by study design, heterogeneity, and quality, reflecting the authors’ qualitative judgment rather than a formal GRADE rating.

## 5. Conclusions

Although heart transplant recipients show a higher rate of general medical complications compared to the general population, the currently available evidence suggests that total joint arthroplasty can be performed with acceptable outcomes in carefully selected patients. The certainty of this evidence remains limited by the retrospective design, overlapping cohorts, and general heterogeneity of the available literature. The mortality events observed in this review were mostly associated with underlying systemic comorbidities rather than the direct surgical procedure. As the survival of heart transplant recipients continues to improve, the demand for joint reconstruction will inevitably rise. TJA remains a vital intervention for relieving pain and restoring function in patients with end-stage joint arthritis. However, achieving the best possible outcomes requires a rigorous approach. We advocate for careful patient selection, comprehensive preoperative counseling regarding the specific risk profile, and a coordinated team including orthopaedic surgeons, anesthesiologists, and transplant physicians to manage the complex perioperative needs of this population. Finally, in order to strengthen the current evidence base and move beyond the limitations of retrospective series, future research directions must prioritize prospective multicenter cohorts and registry-based studies. These future investigations should implement standardized outcome reporting, longer-term follow-up intervals, and rigorous statistical adjustments for relevant clinical confounders to provide more definitive clinical guidelines for this unique patient population.

## Figures and Tables

**Table 1:** Complete List and Results Summary of the Included Studies.

Author (Year)	Study Design	Procedure	Heart Patients (*n*)	Mean Approx. Age (Yrs)	Mean Approx Follow-Up	Main Findings (Heart Specific)
**Agarwal (2022)** [6]	Retrospective (Database)	THA	459	**56**	2 Years	High cardiac complications (32%) and transfusions (14.6%), 0% acute PJI but higher 2-year revision.
**Leonard (2012)** [7]	Retrospective Series	THA/TKA	9	**50**	7.3 Years	0% infection, good functional scores, 20% revision rate (THA).
**León (2007)** [8]	Retrospective Series	THA	18	**52**	35.4 Months (~3 Yrs)	0% revision/infection, high rate of heterotopic ossification (15.8%).
**Hatta (2020)** [9]	Retrospective Cohort	Shoulder	3	**59**	39 Months (3.25 Yrs)	0% infection, Heart patients avoided the fracture complications seen in kidney/liver patients.
**Klement (2016)** [10]	Retrospective (Medicare database)	TKA	412	**<65**	3.5 Years	Highest risk of periprosthetic fracture (OR 8.8) among all transplants, but only at 3 months
**Hsiue (2022)** [11]	Retrospective (Database)	THA/Hemi	170	**50 s**	In-Hospital/90 Days	High mortality/transfusion (including bone marrow recipients), high medical risk.
**Chalmers (2016)** [12]	Retrospective Cohort	THA	18	**59**	4.8 Years	94% implant survival at 5 years, 1 deep infection (5.6%)
**Wu (2022)** [13]	Retrospective Cohort	THA/TKA	19	**56**	2.4 Years	Lowest mortality & transfusion rates among all transplant types.
**Simon (2025)** [14]	Retrospective Cohort	THA	7	**58**	4.6 Years	0% reoperations, 83% 5-year patient survival (better than lung).
Cosic (2019) [15]	Retrospective Series	THA	4	**54**	12 Months	1 intraoperative fracture, generally good functional outcomes at 1 year.
Klatt (2013) [16]	Retrospective Cohort	TKA	4	**60**	7.1 Years	0% infection in non-renal group (incl. heart), significantly lower risk than kidney recipients.
Ledford (2014) [17]	Retrospective Series	THA	4	**64.3**	30 Months	0% infection, highest functional improvement of all groups.
Palmisano (2017) [18]	Retrospective Series	THA	2	**54.8**	30 Months (2.5 Yrs)	0% infection, complications statistically similar to other organ types.
Rizk (2020) [19]	Retrospective Cohort	Shoulder	3	**57.8**	4.0 Years	High medical complications/readmission, no infections reported.
Brown (2020) [20]	Retrospective Cohort	Mixed	20	**59**	7.18 Years	Higher mortality/rehab discharge, infection/readmission same as controls.
Quinlan (2022) [21]	Retrospective (Database)	THA	*-*	**<64–69**	1 Year	Non-renal group had lower revision rate but higher ER visits than controls.
Cavanaugh (2015) [22]	Retrospective (Database)	Mixed	*-*	**58**	In-Hospital	High risk of Acute Renal Failure & Respiratory complications.
Navale (2017) [23]	Retrospective (Database)	THA	*-*	**53.2**	In-Hospital	Higher length of stay, cost, and transfusion risk (SOT aggregate).
Klika (2015) [24]	Retrospective (Database)	TKA	*-*	**60 s**	In-Hospital	Higher cost, length of stay, and complication odds (SOT aggregate).

**Table 2:** The results of the MINORS assessment in detail, indicating Good and Fair methodological quality of the included studies. Key: 0 = not reported, 1 = reported but inadequate, 2 = reported and adequate.

	Item 1	Item 2	Item 3	Item 4	Item 5	Item 6	Item 7	Item 8	Item 9	Item 10	Item 11	Item 12	Total
**Comparative**													
Agarwal (2022) [6]	2	2	0	2	2	2	2	0	2	2	2	2	20
Brown (2020) [20]	2	2	0	2	1	2	1	0	2	2	0	2	16
Cavanaugh (2015) [22]	2	2	0	2	2	2	2	0	2	2	0	2	18
Chalmers (2016) [12]	2	2	0	2	1	2	2	0	1	2	1	2	17
Hatta (2020) [9]	2	2	0	2	2	1	0	0	2	2	1	2	16
Hsiue (2022) [11]	2	2	0	2	2	2	2	0	2	2	2	2	20
Klatt (2013) [16]	2	2	0	2	1	2	0	0	2	2	1	2	16
Klement (2016) [10]	2	2	0	2	2	2	2	0	2	2	1	2	19
Klika (2015) [24]	2	2	0	2	2	2	2	0	2	2	2	2	20
Ledford (2014) [17]	2	1	0	2	2	2	2	0	0	1	1	2	15
Navale (2017) [23]	2	2	0	2	2	2	2	0	2	2	2	2	20
Quinlan (2022) [21]	2	2	0	2	2	2	2	0	2	2	2	2	20
Rizk (2020) [19]	2	1	0	2	1	2	0	0	2	2	2	2	16
Simon (2025) [14]	2	1	0	2	2	2	1	0	0	1	1	2	14
Wu (2022) [13]	2	1	0	2	2	2	1	0	0	1	1	2	14
**Non-Comparative**													
Cosic (2019) [15]	2	1	0	2	2	2	1	0	-	-	-	-	10
Leonard (2012) [7]	2	2	0	2	1	2	2	0	-	-	-	-	11
León (2007) [8]	2	2	0	2	1	2	2	0	-	-	-	-	11
Palmisano (2017) [18]	2	1	0	2	1	2	1	0	-	-	-	-	9

Note: The methodological quality of each included study should be taken into account when interpreting the findings summarized in this review. Conclusions drawn predominantly from studies of lower (‘Fair’) methodological quality warrant more cautious interpretation than those supported by consistently higher-quality (‘Good’) evidence. For classification purposes, studies were considered to be of ‘Good’ methodological quality if they achieved at least 70% of the maximum possible MINORS score (≥17/24 for comparative studies and ≥12/16 for non-comparative studies), and of ‘Fair’ quality if they scored below this threshold (50–69% of Max. MINORS score).

## Data Availability

The data supporting the findings of this study are available from the corresponding author upon reasonable request.

## Supplementary Materials

The following supporting information can be downloaded at: https://www.mdpi.com/article/10.3390/jcm15176794/s1, PRISMA 2020 Checklist. Reference [32] is cited in the Supplementary Materials.

## Abbreviations

The following abbreviations are used in this manuscript:

PJI	periprosthetic joint infection
SOT	solid organ transplant
TJA	total joint arthroplasty
TKA	total knee arthroplasty
THA	total hip arthroplasty
TSA	total shoulder arthroplasty
